# The Corrosion Resistance of Reinforced Lightweight Aggregate Concrete in Strong Brine Environments

**DOI:** 10.3390/ma15227943

**Published:** 2022-11-10

**Authors:** How-Ji Chen, Yung-Chieh Chen, Chao-Wei Tang, Xuan-Fan Lin

**Affiliations:** 1 Department of Civil Engineering, National Chung-Hsing University, 145 Xingda Road, South District, Taichung City 40227, Taiwan; 2 Department of Civil Engineering and Geomatics, Cheng Shiu University, No. 840 Chengching Road, Niaosong District, Kaohsiung 83347, Taiwan; 3 Center for Environmental Toxin and Emerging-Contaminant Research, Cheng Shiu University, No. 840 Chengching Road, Niaosong District, Kaohsiung 83347, Taiwan; 4 Super Micro Mass Research and Technology Center, Cheng Shiu University, No. 840 Chengching Road, Niaosong District, Kaohsiung 83347, Taiwan

**Keywords:** lightweight aggregate, lightweight aggregate concrete, marine corrosion, floating foundation

## Abstract

Taiwan has used technology in reservoir sediments and industrial waste to produce high-performance lightweight aggregate (LWA). LWA can be used to manufacture lightweight aggregate concrete (LWAC) with structural strength ratings. At present, Taiwan’s offshore wind turbines are gradually developing and are moving from coastal areas to deep-sea areas. With this in mind, this study aimed to investigate the feasibility of applying LWAC with synthetic LWA from reservoir sediments to floating offshore wind turbine foundations. LWAC and normal-weight concretes (NWC) of different strengths were prepared, and their fresh, hardened, and durability properties were tested. In addition, reinforced concrete and steel sheets were immersed in a tank of high salinity seawater to examine their resistance to seawater-accelerated corrosion. The test results showed that the total passing charge of the two groups of concrete within six hours was less than 1000 coulombs. Both groups of concrete were classified as having “Very Low” chloride permeability. The average corrosion potential of most reinforced concrete specimens was found to be greater than −200 mV, which means that the corrosion probability of the steel bars was less than 10%. Furthermore, the use of coatings for seawater corrosion protection on steel sheets was not found to be as effective as reinforced concrete. This shows that the use of LWAC with synthetic LWA from reservoir sediments for the floating foundations of offshore wind turbines is feasible and has design flexibility.

## 1. Introduction

In the past twenty years, the development of renewable energy and green energy has become an international focus and trend [1]. Offshore wind power refers to the construction of offshore wind power plants, which are usually installed on the continental shelf and use wind energy to generate electricity. Due to the gradual saturation of offshore wind energy development at onshore and offshore sites, the development of offshore wind turbines in deep water areas has become a future trend. However, with the increase in the seawater depth, the fixed offshore wind turbine foundations used in offshore areas in the past are no longer suitable for deep-sea areas. Therefore, the application of floating offshore wind turbine foundations has become the focus of prospecting in the deep-sea area. Generally, the floating foundation uses the anchoring system to anchor the floating body structure to the seabed and serves as the basic platform for installing the wind turbine. The floating offshore wind turbine foundation needs to withstand the wind turbine and its supporting structure, as well as wave loads [1].

General floating offshore wind turbine foundations are mostly designed with reinforced concrete [2]. However, in marine environments, concrete structures are subject to various factors and can deteriorate [3]. The causes of concrete deterioration can be divided into chemical damage and physical damage. Chemical damage is caused by the hydrolysis or exudation of hard cement, the exchange reaction of harmful factors with hardened cement, or the formation of expansive compounds. Physical damage is divided into two types: wear and cracking. There are many various corrosive ions in seawater, such as Cl^−^, SO_4_^2−^, and Mg^2+^, which can cause the corrosion of concrete structures [4,5]. Among them, Cl^−^ and SO_4_^2−^ have the most serious influences on reinforced concrete. For reinforced concrete structures completely immersed in seawater, the corrosion of sea salt to concrete is mainly a chemical reaction that dissolves calcium hydroxide in the concrete and forms expansive ettringite. This leads to deterioration phenomena such as the swelling, cracking, and spalling of the structure. In addition, the sea level changes with the tide and the alternation of wet and dry periods caused by the change in the sea level will significantly accelerate the deterioration of concrete structures. This behavior is a combination of physical erosion and chemical corrosion. For example, tidal actions can produce waves of different wavelengths and wave heights, which can cause physical impact erosion damage to concrete structures. As for chemical corrosion damage, after salt penetrates the concrete, the crystallization pressure caused by the dry–wet cycle causes the concrete to crack [6]. This leads to corrosion of the rebars in the concrete due to the influence of chloride ions. Moreover, the chemical reaction between sea salt and cement hydration products can form expansive compounds.

Seawater salinity is generally defined as the salt concentration (e.g., sodium and chloride) in seawater [7]. It is measured in PSUs (Practical Salinity Units), which are based on the properties of seawater conductivity. One PSU is equivalent to the concentration per thousand liters, or (‰). In seawater, there is typically close to 35 g of dissolved salts in each liter. Due to the coupling of multiple factors in the marine environment, the durability of many marine concrete structures deteriorates, and their service life is generally less than ten years [8]. In view of this, for a long time, many researchers have been engaged in improving the durability of concrete exposed to high-salt environments, including studies on the effects of single-salt and multi-ion interactions in the marine environment on the durability of concrete.

Poupard et al. [9] conducted a detailed investigation of chloride-induced corrosion damage on a 40-year-old reinforced concrete beam exposed to a marine environment. An analysis of the data showed that, on the beam, two area types could be distinguished: “high corrosion areas” and “low corrosion areas”. “High corrosion zones” were close to corrosion-induced cracks and had different morphologies. Chen et al. [10] evaluated the effect of the curing conditions on the strength, porosity, and chloride intrusion properties of concrete made from blast furnace slag blast furnace cement (HBFC) and ordinary Portland cement (OPC). Their results showed that the early strength of the seawater-cured specimens was slightly higher than that of the air-cured specimens, but the ultimate strength was lower. The chloride diffusion coefficient of HBFC concrete was much lower than that of the corresponding OPC concrete; that is, the resistance of HBFC concrete to chloride ion penetration was significantly better than that of OPC concrete. Medeiros et al. [11] analyzed the chloride ion content of the pillars of reinforced concrete structures exposed to a marine environment, about 700 m from the coastline, for 40 years. The results showed that, in the absence of wetting and drying cycles, the chloride ion content of the pillars was less than the threshold limit of 0.40% per mass of cement. Lei et al. [12] proposed a method for the preparation of superhydrophobic concrete that was non-toxic, corrosion-resistant, and mechanically stable. The results showed that the water contact angle and water slide angle of the prepared superhydrophobic concrete reached 159° and 5°, respectively. Compared with ordinary concrete, the anti-corrosion performance of the prepared superhydrophobic concrete was greatly improved, indicating its important potential application value in marine engineering. Yao and Chen [13] analyzed the damaging effects of various corrosive ions (Cl^−^, SO_4_^2−^, Mg^2+^) on the tensile and compressive strength values of concrete. The quantitative results showed that, during the 600-day corrosion process, the addition of Cl^−^ could weaken the corrosive effect of SO_4_^2−^ by about 20%, while the addition of Mg^2+^ or Mg^2+^ and Cl^−^ could strengthen it by 10–20%.

The pH of seawater varies between 7.4 and 8.4 [14]. In the marine environment, the corrosion of steel bars of reinforced concrete is mainly due to the damage to the alkaline protection provided by the surrounding concrete by the aggressive salts. Among them, the chloride ion has the most serious effect [15], and the degree of its intrusion into the concrete will vary according to the location of the structure. Under the action of tides and waves, the cement concrete in the water level fluctuation area and the splash area repeatedly undergoes the process of being wetted by seawater and water evaporation-drying. This leads to the continuous concentration and precipitation of salt and the growth of crystals, resulting in the destruction of the concrete substrate. Therefore, many researchers have explored the effect of high salinity on the properties of concrete. Wang et al. [16] investigated the durability of reactive powder concrete containing mineral admixtures in seawater erosive environments. The accelerated corrosion test of concrete was carried out using seawater with a concentration of five times the corrosion solution. Kaushik et al. [17] explored the effect of the mixing and curing effect of seawater on concrete durability. The corrosive solution used is five times the concentration of seawater. El-Khoury et al. [18] investigated the effect of cement type and seawater salinity on concrete offshore structures, immersing mortar samples in tap water and two exposure solutions with different salinities (35 g/L and 70 g/L), to examine the effect of increased salinity on the accelerated attack process. The results show that the mass and volume of the hollow cylinders increased, and these evolutions accelerated with increasing salinity. Ikponmwosa et al. [19] simulated two curing media five times (salt solution I) and ten times (salt solution II) the chloride content of lagoon water. The results show that the structural strength of the concrete samples cured in lagoon water was recorded as lower compared to that of the samples cured in salt solution I, but the strength development was higher than that of the samples cured in salt solution II. Uneke et al. [20] studied the effect of salinity on concrete substrates, focusing on magnesium sulfate as the sole attacking agent. The concrete cubes were cured in five different concentrations (0, 5, 10, 15, and 20 g) of magnesium sulfate solution. The results obtained show that the compressive strength of concrete decreased with the increasing amount of magnesium sulfate in the solution. Zhao et al. [21] prepared artificial seawater with one, two, three, and four times the concentration of seawater to analyze the corrosion resistance of concrete. The test results show that, with the prolongation of corrosion age, the volume expansion rate and strength loss of concrete increased rapidly at first and then became stable after reaching a certain age. The erosion of concrete at one times the artificial seawater concentration and two times the artificial seawater concentration had little difference, and the volume expansion rate at three times the artificial seawater concentration increased by 8.6% and 10.6% at 56, 90, 180, and 360 days, respectively. At 0%, 7.2%, and 8.0%, the strength loss rates corresponding to age were 15.1%, 17.1%, 8.4%, and 19.4%, respectively. As an accelerated corrosion test, it is recommended to use three times the concentration of artificial seawater.

The unit weight of lightweight aggregate concrete (LWAC) prepared by utilizing the low-density characteristics of lightweight aggregate (LWA) is lower than that of general concrete. In the early days, LWAC was only used for non-structural applications due to its low compressive strength. However, with the improvement of the properties of artificial LWAs, the compressive strength of LWAC has been significantly improved, and it has been widely used in structural applications. For example, many large marine structures have gradually used reinforced lightweight aggregate concrete. Under the special conditions of seawater corrosion, the use of reinforced lightweight concrete not only reduces the overall weight of the structure but also effectively reduces the permeability of chloride ions and improves the durability of the structure. Thomas and Bremner [22] reported findings on LWAC exposed to oceanic tidal zones. In terms of resistance to chloride penetration, LWAC showed a better-than-expected performance, which may be partly attributed to the “internal cure” provided by LWA, which reduces the effects of self-drying. Wasim and Hussain [23] analyzed the corrosion severity of various types of concrete at elevated temperatures. The test results showed that the LWAC specimens corrode at even lower levels than self-compacting concrete specimens at all exposure temperatures. The reason for this is their higher resistance to chloride attack and the oxygen diffusion provided in their dense matrixes. Real and Bogas [24] investigated the characteristics of chloride entry into structural LWAC in a real marine environment. The experimental results showed that structural LWAC with dense LWA can achieve a chloride permeation resistance equivalent to that of normal-weight concrete (NWC) with the same composition. Real et al. [25] analyzed the penetration of chlorides into bulk structural LWAC after a 5-year exposure period to harsh ocean conditions. The results showed that LWAC exhibited the same durability as NWC. However, despite having the same chloride diffusion coefficient, LWAC tended to have a higher surface chloride content than NWC, resulting in higher chloride permeability at a given depth.

For nearly two decades, Taiwan has developed techniques to use reservoir sediments and industrial waste to manufacture LWAs with excellent performance [26,27,28,29,30,31,32,33]. These LWAs can be used to make LWACs with structural strength ratings. At present, Taiwan’s offshore wind turbines are gradually developing and are moving from the coast to deep-sea areas. If the LWA made in Taiwan is applied to the floating foundation of offshore wind turbines, it will not only solve the problem of the subsequent treatment of Taiwan’s reservoir sediments, but it will also make the design of the foundations of offshore wind turbines more flexible. In view of this, this study aimed to explore the feasibility of applying LWAC with synthetic LWAs from reservoir sediments to floating offshore wind turbine foundations. The main reasons for the corrosion of steel bars in concrete are generally divided into three types: the carbonization of concrete, the depassivation of steel bars caused by chloride ions, and the corrosion of steel bars caused by acid substances [34]. In the composition of seawater, it is known that alkali metal and alkaline earth metal cations have no effect on the corrosion of steel bars, and sulfate ions have an inhibitory effect on the corrosion of steel bars [35]. Therefore, this study mainly considered the influence of chloride ions. Concretes of different strengths from a control group (using normal-weight aggregates) and an experimental group (using light aggregates) were prepared. Through the compressive strength test, ultrasonic pulse velocity test, chloride ion penetration test, electrical indication of concrete’s ability to resist the chloride ion penetration test, steel corrosion potential test, and seawater corrosion resistance test, the differences in the strength, compactness, and durability of the two groups of concrete were analyzed and compared. In corrosive seawater environments, reinforced lightweight concrete not only reduces the overall weight of a structure but also effectively inhibits the permeability of chloride ions, which can improve the durability of the structure.

## 2. Experimental Procedure

### 2.1. Experimental Program

In this study, two groups of concrete were prepared, with lightweight aggregate concrete (LWAC) as the experimental group and normal-weight aggregate concrete (NWC) as the control group. The experimental program used for the two groups of concrete is shown in Table 1. It includes seven main test items. The table also lists the specimen size, the number of specimens, and the test specifications for each test item.

### 2.2. Materials

**Figure 1:**
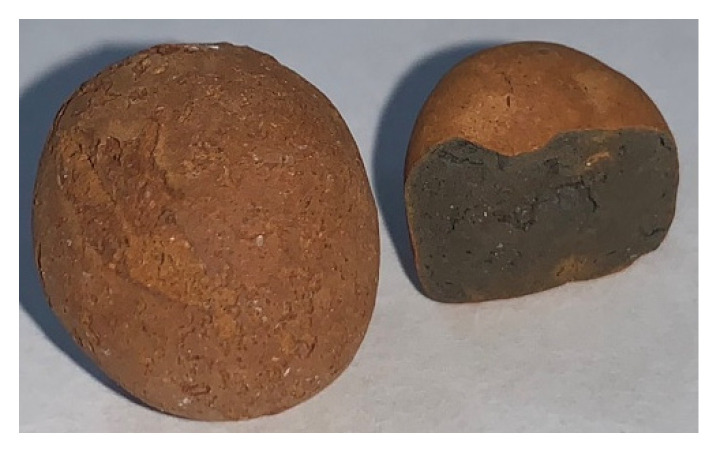
Appearance of lightweight aggregates.

The materials used in this study included cement, ground-granulated blast-furnace slag, silica fume, water, superplasticizer, fine aggregate, coarse aggregate, lightweight aggregate, rebar, and steel sheets. The cement was Portland Type I ordinary cement produced by Taiwan Cement, and its specific gravity was 3.15. The slag was purchased from Jinyu Enterprise, and its specific gravity was 2.8. The specific gravity of the silica fume was 2.2, and its SiO_2_ content was between 86% and 92%. The superplasticizer met the ASTM C494-81 Type F requirements and was a product of Taiwan Sika Corporation (R-550). The fine aggregate was natural sand with a specific gravity of 2.65 and a fineness modulus of 2.8. The coarse aggregate was natural crushed stone with a particle size of about 5 to 10 mm and a specific gravity of 2.65. The lightweight aggregate was an artificial lightweight aggregate, as shown in Figure 1. It met the requirements of ASTM C 330 “Standard Specification for Lightweight Aggregates for Structural Concrete”. The properties of the lightweight aggregates are shown in Table 2. The rebar was locally produced #6 rebar. The steel sheets were carbon steel sheets that were 300 × 200 × 4 mm in size, and there were six pieces in total. They were treated with the following anti-corrosion treatments: electro-galvanizing, hot-dip galvanizing, fluorocarbon baking paint, red lead paint, and RS-65. An original steel sheet was used as the control group. 

### 2.3. Mix Proportions of Concrete

In the concrete mix design, the experimental group and the control group were each formulated with three different compressive strength grades to explore their resistance to seawater corrosion. In order to increase the compactness of the two groups of concrete, part of the cement was replaced with silica fume and slag in each concrete mixture, and the replacement amounts were 8% and 15% of the cement weight, respectively. At the same compressive strength, because the strength of the LWA was lower than that of the natural aggregate, the water–binder ratio of the experimental group was lower than that of the control group. Based on the above scheme, the concrete mix proportions of the experimental group and the control group are shown in Table 3 and Table 4, respectively.

### 2.4. Casting of Specimens

Due to the high-water absorption of LWAs, pre-wetting was performed one day before mixing. The prewetted LWAs were taken out and drained two hours before mixing to allow them to reach a saturated surface-dry condition. The natural fine aggregates and coarse aggregates were wetted by sprinkling water and mixed evenly until a saturated surface-dry condition was attained. 

During the mixing operation, cement, slag, silica fume, and natural sand were first put into a forced single-shaft mixer and mixed for 30 s to achieve uniform mixing. Next, the water (evenly mixed with the superplasticizer) was poured into the mixing cylinder and mixed for 60 to 90 s to make a uniform cement mortar. Then, the light aggregate or coarse aggregate was poured into the mixing cylinder and mixed for about 90 to 120 s to produce uniform fresh concrete. After the mixing had been completed, part of the concrete slurry was taken and used for the slump, chloride ion content, air content, and other tests, and the rest of the concrete slurry was used to cast the specimens for various tests. The size and quantity of the specimens are shown in Table 1. 

**Figure 2:**
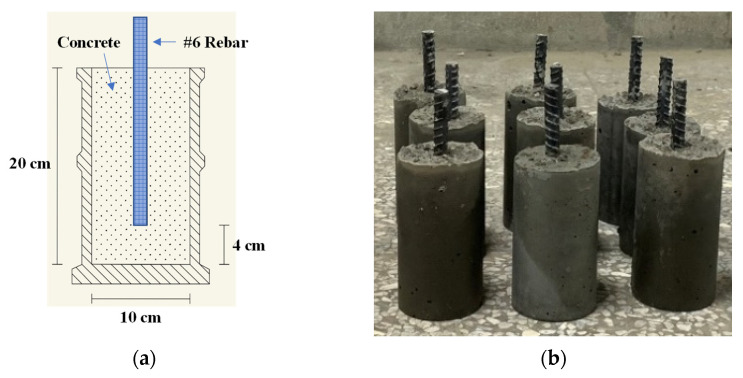
Steel corrosion potential test specimen: (**a**) schematic diagram of the rebar configuration for the steel corrosion potential test specimen; (**b**) epoxy resin coating on the protruding rebars for the steel corrosion potential test specimens.

A cylindrical specimen with a diameter of 10 cm and a height of 20 cm was cast in two layers, while a cylindrical specimen with a diameter of 15 cm and a height of 30 cm was divided into three layers. Each layer was tamped with a tamper and tapped with a rubber mallet to fully compact the specimens. For the specimen used in the steel corrosion potential test, the rebar was fixed in the center of the cylindrical specimen mold with iron wire, 4 cm away from the bottom of the mold, as shown in Figure 2a. The rebar projected a height of 50 mm from the cylinder and acted as an anode. Once the specimens had been cast, they were covered with plastic film and allowed to stand. After 24 h, the specimens were demolded. The protruding rebar of the specimen used in the steel corrosion potential test was coated with epoxy resin, as shown in Figure 2b. Then, the specimens were immersed in a water tank for curing.

### 2.5. Test Methods and Data Analysis

After the specimens in each group had completed 28 days of curing, mechanical, durability, and seawater accelerated corrosion tests were carried out. The methods used for each test are described below.

#### 2.5.1. Compressive Strength Test

Twenty-four hours before the planned compressive test duration, the specimens were taken out of the curing tank or the seawater test tank. In order to reduce the test error, the compression surface of the specimens was ground to a flat surface with a concrete grinder. After grinding, the specimen was allowed to stand for 24 h to allow it to reach an air-dry state. In accordance with the ASTM C39 specification [36], the cylindrical specimen was placed on the compression table in an MTS universal testing machine and was loaded at a compression rate of 0.2 mm per minute until the specimen failed. Then, the ultimate load was divided by the cross-sectional area of the specimen, and this value was taken as the compressive strength.

#### 2.5.2. Elastic Modulus Test

In accordance with the ASTM C469 specification [37], the cylindrical specimen of the compression test was equipped with a strain ring and placed on the compression table of the MTS universal testing machine. A compressive rate of 0.2 mm per minute was applied until the specimen failed. Then, the displacement measured by the strain ring was converted into strain, and the elastic modulus was obtained by dividing the stress value by the strain value.

#### 2.5.3. Ultrasonic Pulse Velocity Test

Specimens that were about to reach the 28-day curing age were taken out of the curing water tank 24 h in advance. Both sides of the cylindrical specimen were ground to a flat surface with a concrete grinder. The purpose of this was to ensure that the contact surface between the ultrasonic tester and the concrete specimen was tightly connected, thereby reducing errors. After grinding, the specimens were allowed to stand for 24 h to reach an air-dry state. The instrument wave velocity was calibrated with a calibration rod according to the ASTM C597 specification [38]. During the test, the pulse end and the receiving end were closely attached to both sides of the concrete specimen. Then, the ultrasonic velocity was determined by dividing the specimen length by the measured time. 

#### 2.5.4. Chloride Ion Penetration Test

**Figure 3:**
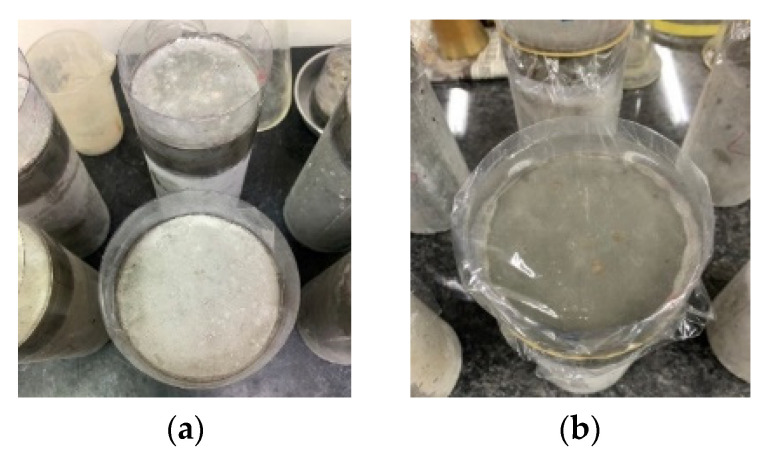
Chloride ion penetration test specimens: (**a**) dike along the perimeter of the top surface of the chloride penetration test specimen; (**b**) specimen with a PE film sealing the opening of the dike.

The chloride ion penetration test was performed in accordance with the ASTM C1543 [39]. After 28 days of curing, epoxy was applied to the sides of the chloride penetration test specimens. A dike with a height of approximately 20 mm was made along the perimeter of the top surface of the specimen, as shown in Figure 3a. A ponding solution was prepared, which was distilled water containing 3% of reagent grade sodium chloride (NaCl) by mass. Then, the surface of the specimen was covered with the ponding solution to a depth of 15 ± 5 mm. In order to prevent the concentration change caused by the evaporation of the solution, the opening of the dike was sealed with a PE film, as shown in Figure 3b. The ponded specimens were stored in an environment of 23.0 ± 2 °C and 50 ± 5% relative humidity. After the immersion age of the specimen reached 60 days, the ponding solution was replaced once. Then, after immersing the specimen for another 30 days, the sample for the chloride ion test of the concrete was obtained by coring with a punching machine. Sampling points were located at four different depths in the specimen to provide a profile of the chloride penetration. The samples were ground into powder, which had to pass through the 0.85 mm test screen. Then, the chloride ion concentration of the samples was measured at each depth with a potentiometric titrator.

#### 2.5.5. Electrical Indication of Concrete’s Ability to Resist the Chloride Ion Penetration Test

**Figure 4:**
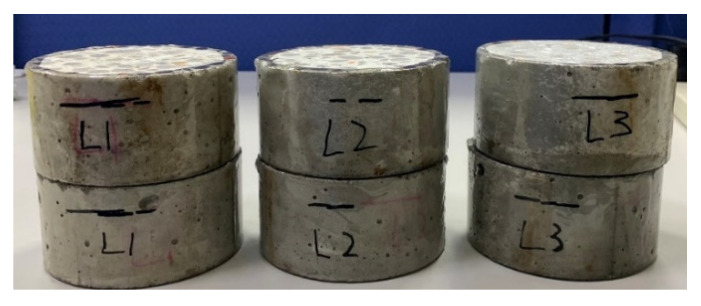
Coating of the side of the experimental group (L1, L2 and L3 mixtures) specimen with fast-setting epoxy resin.

**Figure 5:**
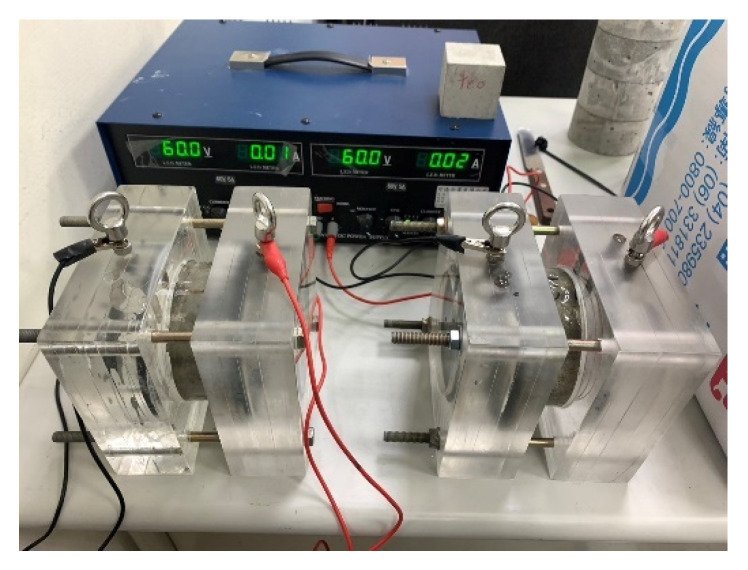
Set-up of the rapid chloride permeability test.

The electrical indication of concrete’s ability to resist the chloride ion penetration test was carried out in accordance with the ASTM C1202-19 specification [40]. After 28 days of curing, the specimens were taken out of the curing water tank and sawed into cylindrical samples with a thickness of 50 mm and a nominal diameter of 100 mm. Next, the samples were placed in the air for at least 1 h to allow their surfaces to dry. Then, the sides of the samples were coated with fast-setting epoxy resin, as shown in Figure 4. The samples were then placed in a vacuum desiccator with an absolute pressure of below 50 mm Hg and left there for 3 h. After that, the samples were immersed in degassed water and continuously operated for 1 h using a vacuum pump. The samples were then continuously immersed in degassed water for 18 h to ensure that their internal pores filled with water. The samples were then placed in a rapid chloride permeability solution, as shown in Figure 5. In addition, 0.3 N sodium hydroxide solution and 3% sodium chloride solution were prepared with distilled water. It should be noted that the heat generated when mixing the sodium hydroxide solution would have affected the conductivity of the solution and the test results. After mixing, the solutions were left to stand. After cooling down, the prepared solutions were put into the test reservoirs on both sides. The test voltage was 60 V, the current value was recorded every 30 min, and the test was completed after 6 h. Integrating the area underneath the current (in amperes) versus time (in seconds) curve yielded the total passing charge (in coulombs) over the 6 h period. 

#### 2.5.6. Seawater Accelerated Corrosion Test

Generally, the normal range of seawater salinity is from 33‰ to 37‰. In order to carry out an effective test in a short period of time, it is often used to increase the concentration of the solution to accelerate the corrosion. In this study, a high-salinity seawater solution with a temperature of 60 degrees Celsius was designed. This makes various compounds in the high-concentration seawater fully soluble to ensure that the seawater has a high content of water-soluble chloride ions. In accordance with the ASTM D1141-98 specification [41], the chemical composition of the substituted ocean water used is shown in Table 5. The salinity was approximately ten times that of normal seawater. The purpose of this was to simulate the accelerated corrosion process of concrete and reinforced concrete specimens in a high-salinity seawater environment in the experimental and control groups. The accelerated corrosion test in seawater mainly explored changes in the compressive strength, microstructure, corrosion potential of steel bars, and corrosion of steel sheets during the accelerated corrosion of the two groups of concrete.

After 28 days of curing, the specimens were immersed in a seawater tank with 10 times the salinity of seawater. After being immersed for the planned durations (28, 90, and 180 days), the specimens were taken out for various tests. The compression test on the cylindrical specimens after immersion in the seawater tank was carried out according to the ASTM C39 specification [36]. There were three specimens in each age group, and the average value was taken. 

**Figure 6:**
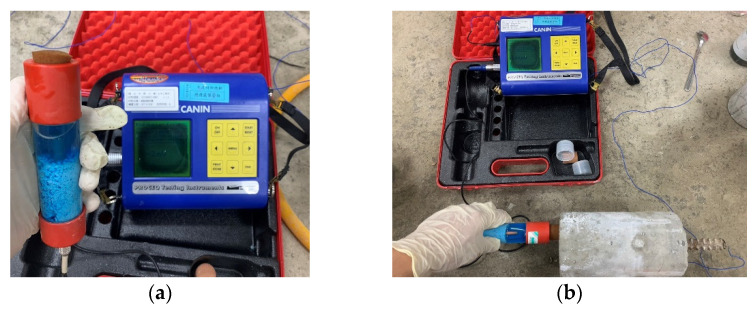
Steel corrosion potential test: (**a**) Copper sulfate solution; (**b**) Setup of the steel corrosion potential measurement.

The test method used to assess the corrosion potential of uncoated reinforced steel in concrete was performed in accordance with ASTM C876 [42]. After the 28-day curing period, concrete cylindrical specimens with embedded reinforcing steel were taken out of the seawater tank. The specimens were then placed in an oven, taken out after two days, and left to cool. A saturated copper sulfate solution was prepared with distilled water and then placed in the joint of the corrosion potentiometer and left to cool, as shown in Figure 6a. Using copper/copper sulfate solution as a reference electrode, the corrosion potentiometer was used to connect concrete and steel bars to generate an electrical circuit. One end of a lead wire was electrically connected to the reference electrode, and the other end of this same lead wire was connected to the negative terminal of the voltmeter. Then, the corrosion potential value of the reinforcing steel embedded in the concrete was detected, as shown in Figure 6b. The potential difference generated by the reduction–oxidation reaction was used to assess the probability of steel corrosion. In the accelerated corrosion test of steel sheets, six steel sheets with dimensions of 300 × 200 × 4 mm were produced. The steel sheets were subjected to anti-corrosion treatments such as electro-galvanizing, hot-dip galvanizing, fluorocarbon baking paint, red lead paint, and RS-65 treated with a corrosion diffusion suppressor, and an original steel sheet was used as a control. It was then placed in a seawater tank with a temperature of 60 degrees Celsius and 10 times the salinity of seawater for accelerated corrosion. In addition, photographs were taken to record the respective rust conditions of the sheets.

## 3. Results and Discussion

### 3.1. Fresh and Basic Mechanical Properties of Concrete

Table 6 shows the test results for the freshly mixed properties of the two groups of concrete. In terms of slump, the slump of each concrete mixture reached more than 15 cm. In addition, the results show that the workability could be increased by pre-wetting the lightweight aggregates and adding superplasticizers. The air content of the L1, L2, and L3 mixtures was 2.9%, 2.8%, and 3.5%, respectively, while the air content of the N1, N2, and N3 mixtures was 2.2%, 3.1%, and 3.6%, respectively. In terms of the chloride ion content, the mixtures of the two groups of concrete both contained 0.001 kg/cm^3^. In terms of the unit weight, the difference between the two groups of concretes could be clearly seen. The fresh unit weights of the L1, L2, and L3 mixtures were 1998, 2007, and 2122 kg/m^3^, respectively, while those of the N1, N2, and N3 mixtures were 2391, 2397, and 2402 kg/cm^3^, respectively. The average fresh unit weight of the experimental group was 15% lower than that of the control group. This shows that the lightweight aggregates used could effectively reduce the unit weight of concrete.

The compressive strength, elastic modulus, and air-dry unit weight of the two groups of concrete are shown in Table 7. In terms of the air-dry unit weight, compared with the control group, it reduced by 11% to 22% in the experimental group. The average air-dry unit weight of the experimental group was 16% lower than that of the control group, and it met the requirements of a general LWAC. The 28-day compressive strengths of the L1, L2, and L3 mixtures were 57.3, 67.2, and 76.6 MPa respectively, while those of the N1, N2, and N3 mixtures were 70.2, 78.8, and 91.8 MPa, respectively. Compared with the 28-day compressive strength (26.7–39.5 MPa) of the LWAC-containing mineral powder produced by Chen et al. [43], the LWAC formulated in this study is of a high-strength grade. The 28-day elastic modulus test results show that the values of the experimental group were about 12% to 13% lower than those of the control group. Likewise, the elastic modulus of the LWAC formulated in this study was significantly higher than that shown in the experimental results (7.37–12.96 GPa) of Chen et al. [43]. In terms of the overall strength, the strength of the experimental group was lower than that of the control group. However, the strength of the experimental group could still reach the level of high-strength concrete. This shows that the lightweight aggregates used had sufficient mechanical strength.

### 3.2. Durability of Concrete

#### 3.2.1. Results of the Ultrasonic Pulse Velocity Test

The ultrasonic pulse velocity test was carried out on the cylindrical specimens that had been cured for 28 days. Two specimens were taken from each concrete mixture for measurement. The test results are shown in Table 8. Ultrasonic waves are diffracted when passing through cracks or pores in the specimen, reducing the transmission speed. Therefore, the compactness of concrete can be estimated using the numerical value of the ultrasonic velocity, which can be determined according to the ASTM C597 specification [38], as shown in Table 9 [44,45]. 

Because the LWA had a higher porosity compared with the natural aggregates, the pulse velocity of the experimental group was lower than that of the control group. In addition, with an increase in the amount of the LWA, the pulse speed of the experimental group decreased. This is consistent with the findings of Satpathy et al. [46]. The ultrasonic velocity of the experimental group was between 4294 and 4550 m/s, and this value increased as the water–binder ratio decreased. Likewise, the average ultrasonic velocity of LWAC formulated in this study was significantly higher than that shown in the experimental results (3101–3268 m/s) of Chen et al. [43]. The ultrasonic velocity of the control group was between 4441 and 5026 m/s, and this value tended to increase as the water–binder ratio decreased. Overall, reducing the water–binder ratio increased the compactness of the concrete and increased its ultrasonic velocity. According to Table 9, the L1 and L2 mixtures were rated as “good” quality, and the L3, N1, N2, and N3 mixtures were rated as “excellent” quality. This shows that the lightweight aggregates used could formulate LWAC with good compactness.

#### 3.2.2. Results of the Chloride Ion Penetration Test

The test results of the acid-soluble chloride ion concentration in the two groups of concrete samples are shown in Table 10. At the specimen depth of 7.5 mm, the experimental group showed values between 0.654% and 0.872%, while the control group showed values between 0.555% and 0.647%. This shows that the concrete in the experimental group had a higher chloride ion concentration than the control group concrete. By multiplying the chloride ion concentration by the unit weight of a concrete specimen, the chloride ion content in the concrete specimen was obtained, as shown in Table 11. The results suggest that the content of acid-soluble chloride ions in the concrete of the experimental group was not higher or even lower than that of the concrete of the control group. This is because the unit weight of the experimental group was lower than that of the control group. 

**Figure 7:**
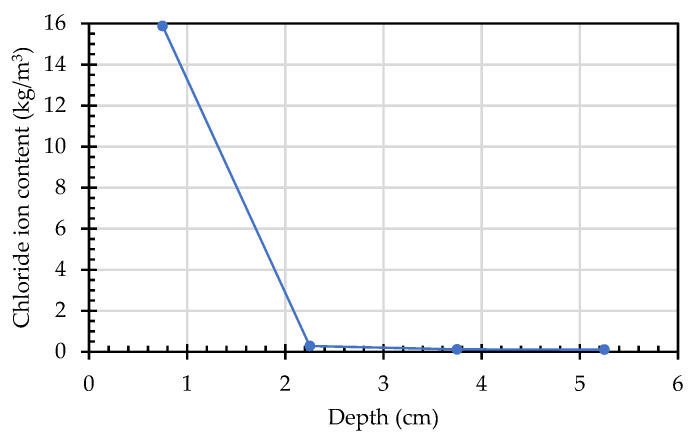
Changes in the chloride ion content of the L1 specimens at different depths.

With the change in the sampling depth, the changing trends in the chloride ion content of each concrete mixture were roughly the same. The chloride ion concentration measured at 22.5 mm for all specimens was significantly reduced compared to that at 7.5 mm. The trace chloride ions measured at 37.5 mm and 52.5 mm could be regarded as chloride ions contained in the concrete itself. Taking the L1 mixture as an example, the chloride ion content was the highest (15.88 kg/m^3^) at the depth of 7.5 mm of the specimen, it was significantly reduced to 0.28 kg/m^3^ at the depth of 22.5 mm, and it dropped to 0.11 kg/m^3^ at depths of both 37.5 mm and 52.5 mm, as shown in Figure 7. In both the experimental group and the control group, chloride ions at depths of greater than 22.5 mm were almost impermeable, and their ability to resist chloride ion penetration was very good. This shows that the lightweight aggregates used could formulate LWAC with good durability. These results are consistent with the chloride ion penetration test results of Liu et al. [47].

#### 3.2.3. Results for the Electrical Indication of Concrete’s Ability to Resist the Chloride Ion Penetration Test

**Figure 8:**
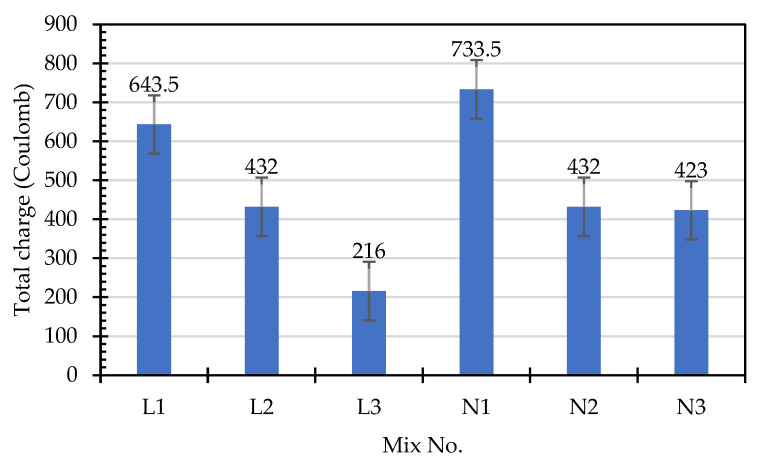
Total charge diagram produced from the chloride ion penetration test.

The chloride ion penetration resistance test results for the concrete specimens in the experimental group and the control group are shown in Table 12 and Table 13, respectively. The average total charge of each mixture is shown in Figure 8. According to the qualitative indication of chloride ion penetration presented in the specification [40], the index of concrete’s resistance to chloride ion penetration can be evaluated, as shown in Table 14. The total passing charge of the experimental group concrete within 6 h was 216–648 coulombs, and the total passing charge of the control group concrete within 6 h was 423–819 coulombs. Compared with the control group, due to the internal curing effect of the experimental group, the porosity of the cement paste was lower, which led to the refinement of the pore structure and thus a reduction in its electric flux [48,49]. The total passing charge of the two groups of concretes within six hours was less than 1000 coulombs. As shown in Table 14, both groups of concretes were classified as having “Very Low” chloride permeability. In other words, their ability to resist chloride ion penetration was very good. This shows that the lightweight aggregates used could formulate LWAC with a good resistance to chloride ion corrosion. According to Liu et al. [50] and Cheng et al. [51], the charge passing through LWAC was 1581–3676 and 461–2153 Coulombs, respectively. In contrast, the charge passing through the experimental group was significantly reduced. Moreover, with a decrease in the water–binder ratios of the two groups of concrete, their compactness increased. The chloride ion penetration test results show that, compared with the concrete in the control group, the concrete in the experimental group performed better in terms of resisting chloride ion penetration. This is consistent with the chloride ion penetration test results.

### 3.3. Results of the High-Salinity Seawater Accelerated Corrosion Test

#### 3.3.1. Compressive Strength of Concrete after the Accelerated Corrosion Test

**Figure 9:**
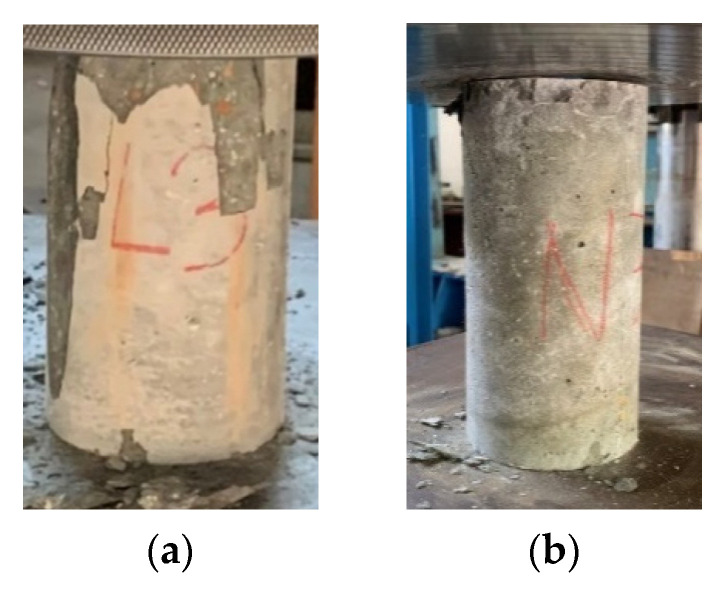
Failure modes of the concrete specimens in the compressive test: (**a**) experimental group (the L3 mixture); (**b**) control group (the N3 mixture).

**Figure 10:**
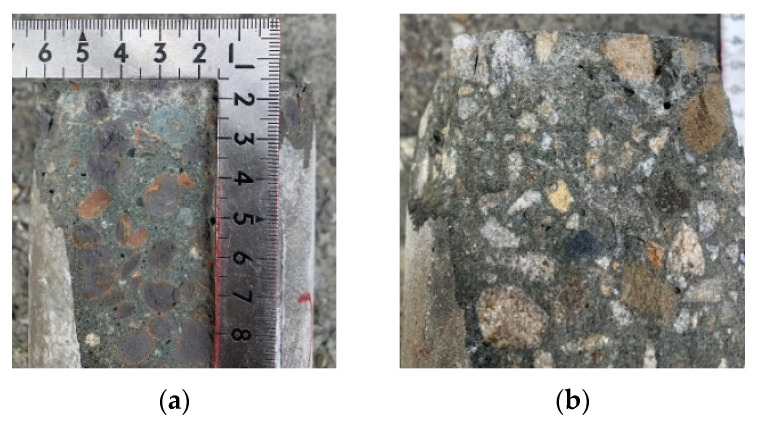
White crystals in the failed concrete specimens: (**a**) experimental group; (**b**) control group.

After completing the standard curing protocol for 28 days, the concrete specimens from the experimental group and the control group were immersed in the high-salinity seawater tank for an accelerated corrosion test. After being immersed for different durations, they were taken out, and their compressive strength was tested. In both the experimental and control groups, the failure of the concrete specimens under compression was all columnar, rather than common cone, split, and shear failures, as shown in Figure 9. In addition, white crystals were found in the pores of the fractured edge fragments of the two groups of concrete specimens, as shown in Figure 10. However, none of these depths exceeded 22.5 mm. This is consistent with the results of the specimens attained in the chloride ion penetration test. 

**Figure 11:**
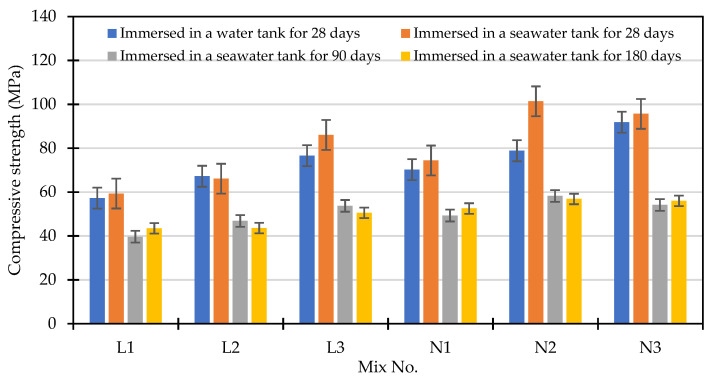
Compressive strength of concrete specimens immersed in a high-salinity seawater tank for different durations.

**Figure 12:**
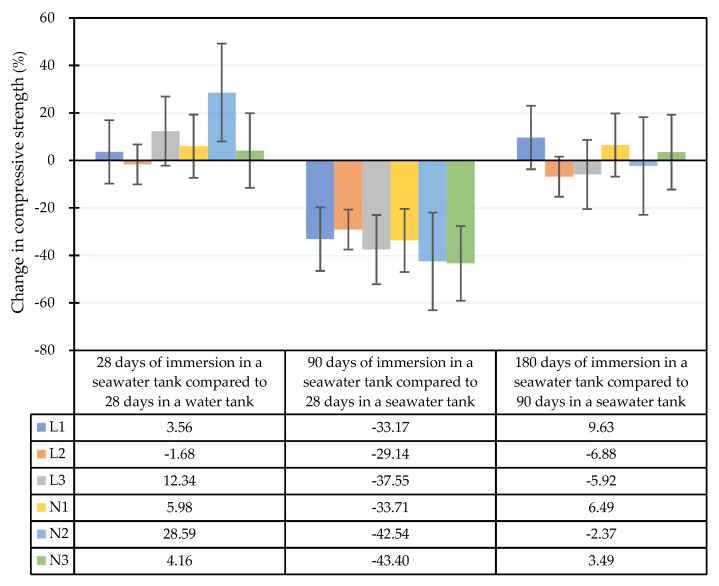
Percentage changes in the compressive strength of concrete specimens immersed in a high-salinity seawater tank for different durations.

The compressive strength test results of each mixture attained from the seawater accelerated corrosion test are shown in Figure 11. Compared with the samples exposed to the accelerated corrosion test for 28 days, the strength of the samples exposed to the accelerated corrosion test for 90 days showed a huge decrease of about 29–43%. However, after 180 days of seawater immersion, the specimen strength did not change significantly. In addition, the percentage changes in the compressive strength of the concrete specimens immersed in a high-salinity seawater tank for different durations are shown in Figure 12. After being immersed in a seawater solution for 28 days, the internal hydration reaction had not completely reacted since the actual total age of the specimen was 56 days. Other than the L2 mixture, in which the compressive strength was reduced by 2%, the strength of the other mixtures was about 4–29% higher than the standard curing strength. This is attributed to the fact that the NaCl, MgCl_2_, and CaCl_2_ components in seawater accelerate the hydration reaction of unhydrated cement [51,52]. After being immersed in the seawater solution for 90 days, the compressive strength of each mixture decreased by 29–43% compared with the compressive strength of the samples immersed in the seawater solution for 28 days. After 180 days of immersion in the seawater solution, the compressive strength of each mixture did not change significantly compared with that when immersed in the seawater solution for 90 days, and the changes ranged from a decrease of 9% to an increase of 10%. In general, the strength of each mixture was still greater than the standard of general high-strength concrete (41.2 MPa).

**Figure 13:**
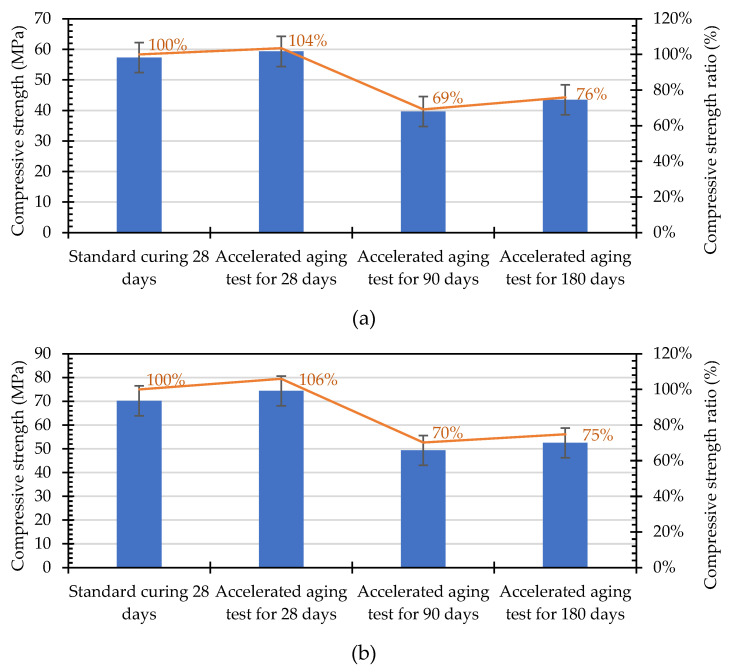
Changes in the compressive strength ratios of concrete specimens with the time of immersion in seawater: (**a**) L1; (**b**) N1.

The compressive strength ratio can be obtained by dividing the compressive strength of the specimen at different accelerated corrosion ages by its compressive strength after curing for 28 days under standard conditions. Taking the mixture with the lowest compressive strength in the two groups of concrete as an example, the compressive strength ratio of each specimen varied with the time of immersion in seawater, as shown in Figure 13. After 180 days of exposure to the seawater accelerated corrosion test, the compressive strength ratio of the L1 mixture was 76%, and the compressive strength ratio of the N1 mixture was 75%.

Based on the above analysis, it can be seen that the seawater deteriorated the surface of the concrete specimen and reduced its strength. However, this deterioration was limited to the surface layer. Therefore, the effect of seawater on concrete strength was limited. The specimen used in this research was a cylindrical specimen with a diameter of 10 cm and a height of 20 cm. If the penetration could only reach about 22.5 mm, the area affected by seawater accounted for more than half of the specimen’s volume. If a cylindrical specimen with a diameter of 15 cm and a height of 30 cm had been used instead, the percentage of the area affected by seawater compared with the overall volume would have been lower. Theoretically, the percentage change in overall strength will not be higher than that of a cylindrical specimen of the current size.

#### 3.3.2. SEM Observation of Concrete after the Accelerated Corrosion Test

**Figure 14:**
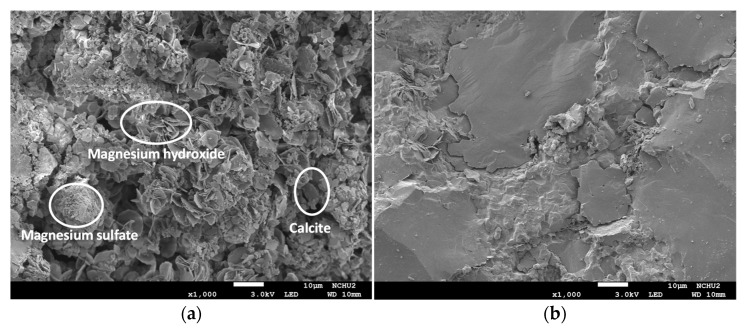
SEM micrographs of the control group specimens magnified 1000 times after the accelerated corrosion test: (**a**) at 7.5 mm; (**b**) at 37.5 mm.

**Figure 15:**
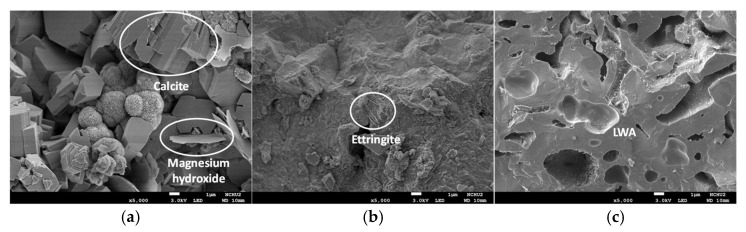
SEM micrographs of the concrete specimens magnified 5000 times after the accelerated corrosion test: (**a**) control group at 7.5 mm; (**b**) control group at 37.5 mm; (**c**). experimental group at 37.5 mm.

After the two groups of concrete specimens had been subjected to the seawater accelerated corrosion test, the internal interface transition zone (ITZ) and pore structure were observed by scanning electron microscopy. Because the 28-day compressive strengths of the L3 and N2 mixtures were similar (76.59 and 78.83 MPa, respectively), samples were taken from the compressive test pieces after the seawater corrosion of these two mixtures for SEM observation. Compared with the control group, the water in the LWA in the concrete of the experimental group contributed to internal curing, resulting in a higher degree of cement hydration [48,49]. In particular, the cement paste was firmly bonded to the porous LWA surface, and portions of the hydration products filled the pores of the outer layer. In other words, the interface between the LWA and hardened cement paste is not obvious. This is consistent with the results of Cheng et al. [51]. Therefore, the microstructures of the two groups of concrete were different. Figure 14 shows 1000-times magnified photos of the concrete specimens from the control group at depths of 7.5 mm and 37.5 mm. An obvious difference in the overall structures of the two places can be observed. The sample at 7.5 mm contained petal-shaped magnesium hydroxide, irregular block-shaped magnesium sulfate, and columnar calcite, while the above-mentioned phenomenon was not observed for the sample at 37.5 mm. Figure 15 is an SEM photograph of a concrete sample magnified by 5000 times. The columnar structure of calcite in the concrete sample of the control group at a depth of 7.5 mm became more obvious, and there were more spherical structures. However, this phenomenon was not observed in the two groups of concrete samples at a depth of 37.5 mm, and some needle-like ettringite and massive C-H crystals were observed on the surface of the control group.

**Figure 16:**
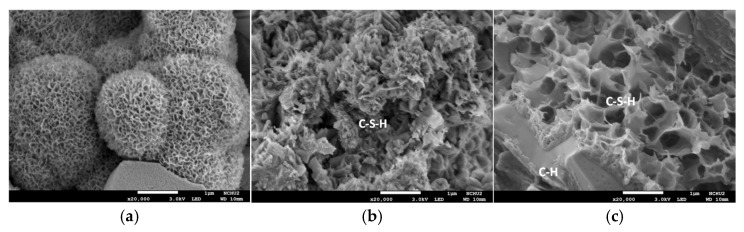
SEM micrographs of the concrete specimens magnified 20,000 times after the accelerated corrosion test: (**a**) control group at 7.5 mm; (**b**) control group at 37.5 mm; (**c**). experimental group at 37.5 mm.

An SEM photo of the sample magnified 20,000 times is shown in Figure 16. The spherical structure became quite evident at 7.5 mm for the control specimen. C-S-H crystals (irregular layered structure) were observed at a depth of 37.5 mm in the two groups of specimens. The above analysis echoes the experimental results presented in the previous section. That is to say, the effect of the accelerated corrosion test on concrete was reflected in its surface layer, so there were significant microstructural changes (such as the formation of spherical, columnar, and other products) at a depth of 7.5 mm. At a depth of 37.5 mm, the same state as general concrete was shown, and chloride ions did not significantly affect the composition of the concrete microstructure.

#### 3.3.3. Corrosion Potential of the Rebar after the Accelerated Corrosion Test

The average steel corrosion potentials of the specimens in each group after being immersed in the seawater tank for different durations are shown in Table 15. In accordance with the relationship between the steel potential value and its corrosion probability (as shown in Table 16 [42]), the average corrosion potential of the L1 mixture immersed for 90 days was −237 mV, and the corresponding corrosion probability was between 10% and 90%. The average corrosion potential of the rest of the specimens was greater than −200 mV, and the corrosion probability was less than 10%. Compared with the potential values of LWAC presented by Wasim and Hussain [23], which ranged from −605 to −596 mV, the corrosion probability of the LWAC formulated in this study was significantly lower.

**Figure 17:**
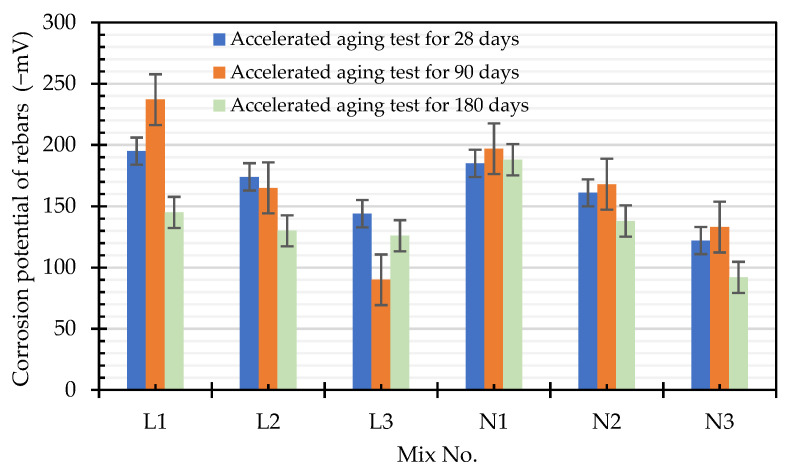
Bar chart of the corrosion potential of the steel bar after the accelerated corrosion test.

**Figure 18:**
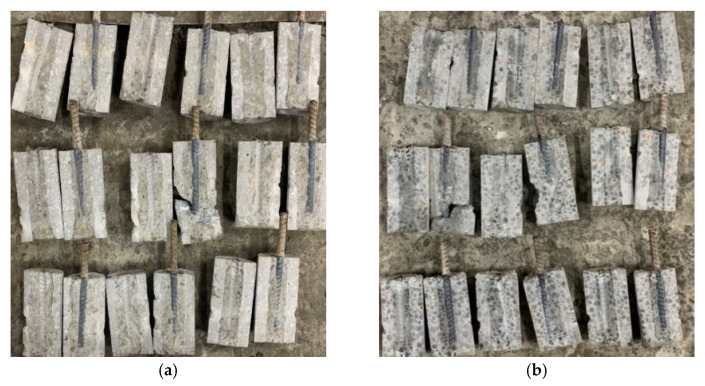
Longitudinal sections of the specimens after 180 days of immersion: (**a**) control group; (**b**) experimental group.

**Figure 19:**
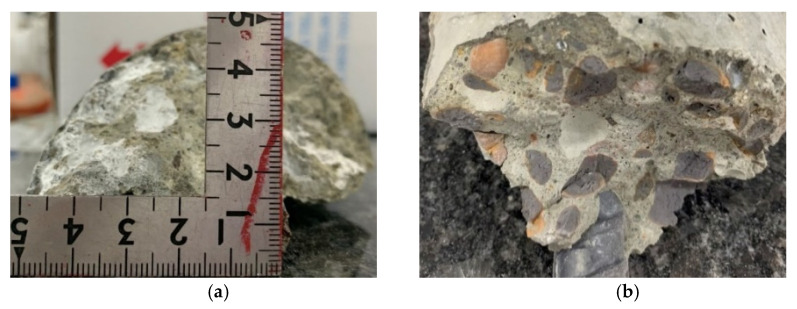
Salt crystals in the specimens after 180 days of immersion: (**a**) control group; (**b**) experimental group.

The corrosion potentials of the two groups of specimens showed a downward trend with an increase in the water–binder ratio. Overall, the corrosion potential of a given specimen did not change significantly after different immersion durations, as shown in Figure 17. In addition, after the specimen was cut along its longitudinal axis, there was no sign of corrosion in its steel bars, as shown in Figure 18. In other words, the two groups of concrete effectively protected the steel bars from corrosion. It is worth noting that, when examining the cross-section of the specimen with an immersion duration of 180 days, it was observed that salt crystals penetrated through the ITZ of the coarse aggregate and the cement paste and the pores of the paste. However, the depths were not more than 22.5 mm, and there were more salt crystals in the ITZ of the control group concrete than in the experimental group concrete, as shown in Figure 19.

#### 3.3.4. Corrosion of Steel Sheets after the Accelerated Corrosion Test

**Figure 20:**
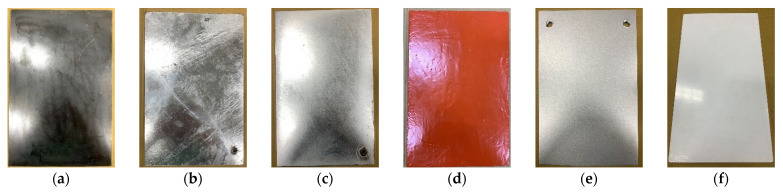
Steel sheets used in the accelerated corrosion test: (**a**) original steel sheet; (**b**) hot-dip galvanized sheet; (**c**) electro-galvanized sheet; (**d**) red lead paint sheet; (**e**) RS-65 sheet; (**f**) fluorocarbon-baked sheet.

The coating film thickness of the steel sheet used in the accelerated corrosion test is shown in Table 17. Photos of the uncoated steel sheet and the steel sheet subjected to different treatments to complete the anti-corrosion coating are shown in Figure 20.

**Figure 21:**
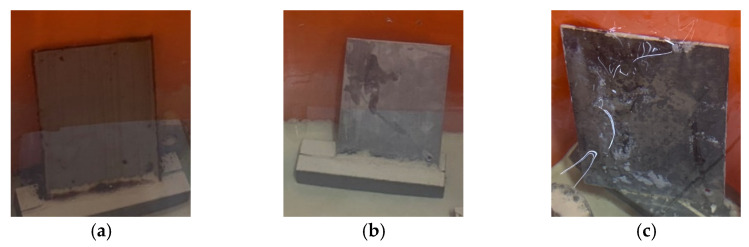
Appearance of and damage done to the steel sheets: (**a**) original steel sheet after 11 days of immersion; (**b**) hot-dip galvanized sheet after 11 days of immersion; (**c**) electro-galvanized sheet after 21 days of immersion.

**Figure 22:**
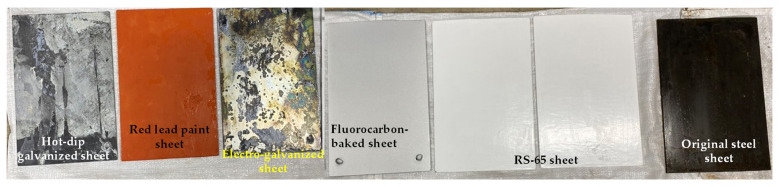
Appearances of the steel sheets after 27 days of immersion.

On the 11th day of the test, the surface layer of the uncoated steel sheet was found to have obvious rust, peeling, and damage, as shown in Figure 21a, while the hot-dip galvanized steel sheet had a large amount of zinc oxide on the surface of the coating, as shown in Figure 21b. On the 21st day of the test, protrusions and a little peeling were also found on the coating surface of the electro-galvanized steel sheet, as shown in Figure 21c. On the 27th day of the test, the red lead paint sheet had a bulge on the surface of the coating, and the coatings on the RS-65 sheet and the fluorocarbon baking paint sheet were not damaged, as shown in Figure 22.

**Figure 23:**
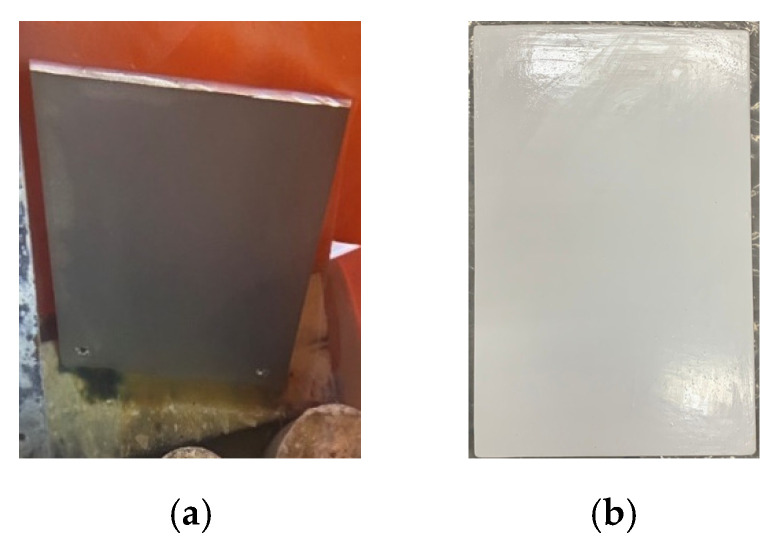
Appearance of and damage done to the steel sheets: (**a**) f fluorocarbon-baked sheet after 40 days of immersion; (**b**) RS-65-treated sheet after 74 days of immersion.

**Figure 24:**
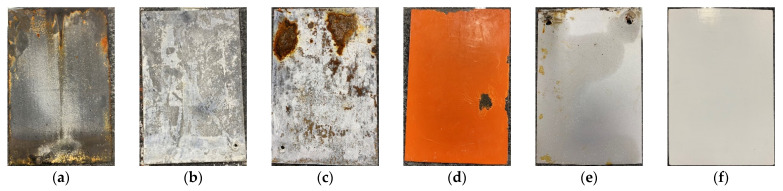
Appearance of the steel sheets after 90 days of immersion: (**a**) original steel sheet; (**b**) hot-dip galvanized sheet; (**c**) electro-galvanized sheet; (**d**) red lead paint sheet; (**e**) f fluorocarbon-baked sheet; (**f**) RS-65 sheet.

On the 40th day, granular protrusions were found on the surface of the fluorocarbon-baked steel sheet, as shown in Figure 23a. On the 74th day, granular protrusions were found on the surface of the RS-65 steel sheet, as shown in Figure 23b, and the other steel sheets were found to have coating bulges or sections that were peeling off. On the 90th day, the coating of the steel sheets was scraped off with a scraper to observe whether there were any signs of damage or corrosion diffusion inside, and the findings were compared with the original steel sheet, as shown in Figure 24. On the 11th day, zinc oxide was observed on the surface of the hot-dip galvanized steel sheet. On the 90th day, the surface conditions continued to deteriorate, and the chalking condition was severe. However, after the surface layer had been removed, no corrosion was observed in the inner steel, as shown in Figure 24b. The electro-galvanized steel sheet not only suffered surface damage but also had internal steel corrosion, as shown in Figure 24c. The coating of the red lead paint sheet was too thick due to self-painting. Only the surface of the coating bulged, but there was no sign of corrosion inside, as shown in Figure 24d. Partial cracking of the coating was found on both the front and back of the fluorocarbon-baked steel sheet. After scraping, there was some corrosion inside, but no signs of corrosion diffusion were observed, as shown in Figure 24e. The RS-65 steel sheet was the slowest sample to experience coating damage. After the coating was removed, only a little damage at the edge was observed, and no signs of corrosion diffusion were found, as shown in Figure 24f.

Overall, the use of coatings for seawater corrosion protection on steel is not as effective as using reinforced concrete. Taking electro-galvanizing as an example, not only is the coating damaged, but the internal steel is also corroded, and the corrosion spreads. Taking hot-dip galvanizing as an example, although only the surface of the steel is damaged, the large amount of zinc oxide powder produced may have an impact on the environment. Furthermore, the appearance is relatively unsightly. In contrast, using concrete as the protective layer of steel means that there is no need to worry about seawater corrosion and other related issues.

### 3.4. Feasibility Study on the Use of Lightweight Aggregates for Offshore Wind Turbine Foundations

The corrosion potential of steel bars and the accelerated corrosion test results of steel sheets show that reinforced concrete provides better protection against the seawater corrosion of steel than the steel anti-corrosion coating treatments used in this study. For example, after the 180-day seawater accelerated corrosion test for each concrete mixture, the steel bars of all reinforced concrete specimens were not corroded. However, all steels with anti-corrosion coating were damaged after 90 days of accelerated corrosion testing. In particular, the steel sheet treated by electro-galvanizing had not only a destroyed coating but also inner steel corrosion. While the hot-dip galvanized steel sheet did not show corrosion of its internal steel, the coating produced a large amount of zinc oxide powder. If this material is used in marine structures, it may have a certain impact on the environment. In contrast, if reinforced concrete is used, this problem is insignificant.

In marine structures, the differences between the use of lightweight aggregates and natural aggregates for reinforced concrete can be divided into physical properties and durability. The main physical properties are the concrete’s compressive strength and unit weight, because different designs have different target strengths. In the concrete mix design used in this study, although the compressive strength of the experimental group was lower than that of the control group, the strength of the L3 mixture reached 76.6 MPa. With a target strength of 70 MPa, using the L3 mixture and the N1 mixture, the compressive strength values reached 76.6 MPa and 70.2 MPa, respectively, meeting the requirements for use. In particular, the unit weight of the experimental group was reduced by 10% compared with that of the control group. In other words, for a given volume, foundations designed with LWAC have a greater buoyancy. In terms of durability, according to the chloride ion penetration test results, the salt crystals were only able to penetrate about 22.5 mm. The results of the rapid chloride permeability test show that, under the condition of a similar water–binder ratio, the ability of LWAC to resist charge intrusion was better than that of NWC. In the seawater accelerated corrosion test, when the compressive strength after 28 days of curing was used as the benchmark, the L1 mixture was the best after 180 days of accelerated corrosion, and the compressive strength ratio after 180 days of accelerated corrosion was 76%. The worst results were produced for the N3 mixture, which had a compressive strength ratio of 61%. 

In view of the above results, for a given compressive strength of concrete, the use of LWA can effectively reduce the unit weight. In terms of durability, the results of the experimental group were no worse, or were better, than those of the control group. Therefore, the use of LWAC for the floating foundations of offshore wind turbines is feasible and has design flexibility.

## 4. Conclusions

This study explored the feasibility of applying LWAC with synthetic LWA from reservoir sediments to the foundations of floating offshore wind turbines. According to the above test results and analysis, the following conclusions were obtained.

- The ultrasonic velocity of the experimental group was between 4294 and 4550 m/s, and this value increased with a decrease in the water–binder ratio. The L1 and L2 mixtures were rated as being of “good” quality, and the L3 mixture was rated as being of “excellent” quality.
- The chloride ion concentration measured at 22.5 mm for all specimens was significantly reduced compared with that at 7.5 mm. Chloride ions at depths of greater than 22.5 mm were almost impermeable, which shows their excellent resistance to chloride ion penetration.
- The total charge passing through the two groups of concrete within six hours was less than 1000 coulombs. Both groups of concrete were classified as having “Very Low” chloride permeability.
- Compared with the accelerated corrosion test age of 28 days, the compressive strength of the accelerated corrosion test age of 90 days had a huge decrease of about 29–43%. However, the compressive strength of the specimens did not change significantly after being immersed in seawater for 180 days.
- The water in the LWA in the concrete of the experimental group contributed to the internal curing, resulting in a higher degree of cement hydration. The cement paste was firmly bonded to the porous LWA surface, and a portion of the hydration product filled the pores of the outer layer.
- The average corrosion potential of the L1 mixture immersed for 90 days was −237 mV, and the corresponding corrosion probability was between 10% and 90%. The average corrosion potential of the rest of the specimens was greater than −200 mV, and the corrosion probability was less than 10%. In addition, there was no sign of corrosion in its steel bars.
- The use of coatings for seawater corrosion protection on steel sheets is not as effective as the use of reinforced concrete. The use of LWAC for the floating foundations of offshore wind turbines is feasible and has design flexibility.

## Figures and Tables

**Table 1:** Experimental program for the two groups of concrete.

Test Item	Specimen Size	Number of Specimens	Test Specification
Compressive strength test	Cylinders (15 cm × 30 cm)	3	ASTM C39
Elastic modulus test			ASTM C469
Ultrasonic pulse velocity test	Cylinders (10 cm × 20 cm)	2	ASTM C597
Chloride ion penetration test		1	ASTM C1543
Electrical indication of the concrete’s ability to resist the chloride ion penetration test		2	ASTM C1202
Steel corrosion potential test		9	ASTM C876
Steel sheet accelerated corrosion test	300 × 200 × 4 mm	6	-

**Table 2:** Properties of lightweight aggregates.

Test Item	Test Results	Test Specifications
Specific gravity	1.41	ASTM C127
Crushing strength	13.96 MPa	CNS 14779
Sodium sulfate soundness test	0.11%	CNS 1167
24-hour water absorption rate	7.8%	ASTM C127
Dry loose unit weight	850 kg/m^3^	CNS 3691
Loss on ignition	0.05%	CNS 3691

**Table 3:** Mix proportions of the LWAC.

Mix No.	W/B	Water (kg/m^3^)	Cement (kg/m^3^)	Silica Fume (kg/m^3^)	Slag (kg/m^3^)	LWA (kg/m^3^)	FA (kg/m^3^)	SP (kg/m^3^)
L1	0.40	170	327	34	64	668	683	2.34
L2	0.32	175	421	44	82	551	758	3.83
L3	0.25	175	533	55	104	413	845	6.92

Notes: W/B, water–binder ratio; LWA, lightweight aggregate; FA, fine aggregate; SP, superplasticizer.

**Table 4:** Mix proportions of the NWC.

Mix No.	W/B	Water (kg/m^3^)	Cement (kg/m^3^)	Silica Fume (kg/m^3^)	Slag (kg/m^3^)	CA (kg/m^3^)	FA (kg/m^3^)	SP (kg/m^3^)
N1	0.44	180	315	33	61	922	862	2.86
N2	0.36	180	385	40	75	908	796	3.75
N3	0.30	180	462	48	90	880	735	4.80

Notes: W/B, water–binder ratio; CA, coarse aggregate; FA, fine aggregate; SP, superplasticizer.

**Table 5:** Chemical compositions of the substitute ocean water samples used.

Compound	Percentage
Sodium Chloride NaCl	58.49%
Magnesium Chloride MgCl_2_	26.46%
Sodium Sulfate Na_2_SO_4_	9.75%
Calcium Chloride CaCl_2_	2.765%
Potassium Chloride KCl	1.645%
Sodium Bicarbonate NaHCO_3_	0.477%
Potassium Bromide KBr	0.238%
Boric Acid H_3_BO_3_	0.071%
Strontium Chloride SrCl_2_	0.095%
Sodium Fluoride NaF	0.007%

**Table 6:** Fresh properties of the concretes.

Mix No.	Slump (cm)	Air Content (%)	Chloride Concentration (kg/m^3^)	Unit Weight (kg/m^3^)
L1	22.0	2.9	0.001	1998.3
L2	20.5	2.8	0.001	2007.3
L3	19.8	3.5	0.001	2122.1
N1	20.0	2.2	0.001	2391.8
N2	21.0	3.1	0.001	2397.7
N3	17.0	3.6	0.001	2402.0

**Table 7:** Hardened properties of the concretes.

Mix No.	Compressive Strength (MPa)	Elastic Modulus (GPa)	Air-Dry Unit Weight (kg/m^3^)
L1	57.29	23.45	1821
L2	67.22	27.28	1913
L3	76.59	30.77	2073
N1	70.20	30.49	2325
N2	78.83	33.61	2254
N3	91.83	35.51	2326

**Table 8:** Ultrasonic velocity test results for the concretes.

Mix No.	Distance (mm)	Time (μs)			Average Time (μs)	Ultrasonic Velocity (m/s)	Average Ultrasonic Velocity (m/s)
L1	198.21	44.8	45.3	45.4	45.2	4387.6	4340.8
	198.06	46.5	46.2	45.7	46.1	4294.0	
L2	197.43	44.6	43.0	44.0	43.9	4500.7	4475.4
	198.77	45.8	44.2	44.0	44.7	4450.1	
L3	199.52	43.3	44.5	45.0	44.3	4507.2	4528.4
	199.73	44.0	44.3	43.4	43.9	4549.7	
N1	198.33	43.5	43.1	43.5	43.4	4573.3	4513.1
	199.04	44.8	44.0	45.3	44.7	4452.8	
N2	197.36	42.2	41.9	42.1	42.1	4691.6	4566.2
	200.72	45.3	44.6	45.7	45.2	4440.7	
N3	196.86	39.0	39.6	38.9	39.2	5026.2	4883.6
	197.70	41.7	42.1	41.3	41.7	4741.0	

**Table 9:** Ultrasonic wave velocities of concrete and quality judgments.

UPV Range (m/s)	Concrete Quality
More than 4500	Excellent
From 3600 to 4500	Good
From 3000 to 3600	Questionable
From 2100 to 3000	Poor
From 1800 to 2100	Very poor

**Table 10:** Acid-soluble chloride ion concentration in concrete specimens.

Mix No.	Depth from the Surface of the Cylindrical Specimen			
	7.5 mm	22.5 mm	37.5 mm	52.5 mm
L1	0.872%	0.016%	0.006%	0.006%
L2	0.653%	0.008%	0.008%	0.005%
L3	0.654%	0.007%	0.005%	0.005%
N1	0.555%	0.021%	0.006%	0.004%
N2	0.647%	0.012%	0.007%	0.003%
N3	0.558%	0.007%	0.003%	0.003%

**Table 11:** Acid-soluble chloride ion content in concrete specimens (Unit: kg/m^3^).

Mix No.	Depth from the Surface of the Cylindrical Specimen			
	7.5 mm	22.5 mm	37.5 mm	52.5 mm
L1	15.88	0.28	0.11	0.10
L2	12.49	0.15	0.14	0.09
L3	13.54	0.15	0.10	0.09
N1	12.91	0.49	0.13	0.09
N2	14.58	0.26	0.15	0.07
N3	12.99	0.17	0.08	0.06

**Table 12:** Results of the rapid chloride permeability test for the experimental group (Units: Amperes).

Time (min)	Mix No.					
	L1		L2		L3	
0	0.03	0.02	0.02	0.02	0.01	0.01
30	0.03	0.03	0.02	0.02	0.01	0.01
60	0.03	0.03	0.02	0.02	0.01	0.01
90	0.03	0.03	0.02	0.02	0.01	0.01
120	0.03	0.03	0.02	0.02	0.01	0.01
150	0.03	0.03	0.02	0.02	0.01	0.01
180	0.03	0.03	0.02	0.02	0.01	0.01
210	0.03	0.03	0.02	0.02	0.01	0.01
240	0.03	0.03	0.02	0.02	0.01	0.01
270	0.03	0.03	0.02	0.02	0.01	0.01
300	0.03	0.03	0.02	0.02	0.01	0.01
330	0.03	0.03	0.02	0.02	0.01	0.01
360	0.03	0.03	0.02	0.02	0.01	0.01

**Table 13:** Results of the rapid chloride permeability test for the control group (Units: Amperes).

Time (min)	Mix No.					
	N1		N2		N3	
0	0.03	0.03	0.02	0.02	0.01	0.01
30	0.03	0.03	0.02	0.02	0.02	0.02
60	0.03	0.03	0.02	0.02	0.02	0.02
90	0.04	0.03	0.02	0.02	0.02	0.02
120	0.04	0.03	0.02	0.02	0.02	0.02
150	0.04	0.03	0.02	0.02	0.02	0.02
180	0.04	0.03	0.02	0.02	0.02	0.02
210	0.04	0.03	0.02	0.02	0.02	0.02
240	0.04	0.03	0.02	0.02	0.02	0.02
270	0.04	0.03	0.02	0.02	0.02	0.02
300	0.04	0.03	0.02	0.02	0.02	0.02
330	0.04	0.03	0.02	0.02	0.02	0.02
360	0.04	0.03	0.02	0.02	0.02	0.02

**Table 14:** Rapid chloride permeability test ratings.

Charge Passed (Coulombs)	Chloride Ion Penetrability
>4000	High
2000–4000	Moderate
1000–2000	Low
100–1000	Very Low
<100	Negligible

**Table 15:** Corrosion potential of rebars after the accelerated corrosion test (Units: mV).

Mix No.	Immersion Time for the Accelerated Corrosion Test		
	28 Days	90 Days	180 Days
L1	−195	−237	−145
L2	−174	−165	−130
L3	−144	−90	−126
N1	−185	−197	−188
N2	−161	−168	−138
N3	−122	−133	−92

**Table 16:** Relationship between the steel potential value and corrosion probability.

Copper/Copper Sulfate Electrode (mV)	Corrosion Probability
>−200	Less than 10%
−200 to −350	Between 10% and 90%
<−350	More than 90%
<−500	Severe corrosion

**Table 17:** Coating film thickness of steel sheets used in the accelerated corrosion test.

Coating Method	Hot-Dip Galvanized	Electro-Galvanized	Red Lead Paint	RS-65	Fluorocarbon Baking Paint
Film thickness (μm)	85	20	500	40	40

## Data Availability

The data presented in this study are available upon request from the corresponding author.
